# Treatment of Trichiasis by Electrolysis

**Published:** 1884-01

**Authors:** J. Elliot Colburn

**Affiliations:** Surgeon at Central Free Dispensary, Eye and Ear Department; Assistant Surgeon at Illinois State Eye and Ear Infirmary; member of Chicago Society of Otology and Ophthalmology


### ARTICLE VI.

**TREATMENT OF TRICHIASIS BY ELECTROLYSIS.** J. ELLIOT COLBURN, Surgeon at Central Free Dispensary, Eye and Ear Department; Assistant Surgeon at Illinois State Eye and Ear Infirmary; member of Chicago Society of Otology and Ophthalmology.

One of the common causes of diseased cornea is displaced or misdirected cilia. They may be irregular in growth, but one or two hairs sweeping the cornea ; or the whole tarsal border may be covered by a dark and strong, or pale and stunted growth of lashes, causing great irritation of the cornea, loss of epithelial substance, followed by ulceration, inveterate pannus or ulcers, causing prolapsis of the iris, anterior synechia and atrophy of the globe. This abnormal growth of the cilia may be spontaneous or caused by chronic inflammation of the conjunctiva of the margin of the lids as in *tinia tarsi*, or *traumatisms*, as burns, wounds of the eye, etc. Trichiasis or districhiasis may be followed by or complicated with entropium in trachoma. The irregular growth of lashes will cause great irritation, producing excessivelachrymation and photophobia, or sensation of foreign body in the eye.

The diagnosis of districhiasis is easy, but in trichiasis the lashes may be so pale and minute as to escape detection. For this reason it is well in all superficial diseases of the cornea to examine the borders of the lids with a three inch lens and a strong light. The treatment of trichiasis consists in the prominent removal of the displaced lashes and the treatment of such complications as may occur. The methods of treatment described in our text books are quite formidable and not altogether satisfactory, as they result in more or less deformity of the lid and destruction of tissues. The method which we have used in more than fifty cases, twenty-two of which I have been able to observe through periods of from six months to three and a half years, is the use of electrolysis as applied in the removal of hirsuties of the face, as described by Drs. Fox, Hays and others. The instruments necessary are first, a galvanic battery of six or more cells; second, a light needle holder armed with a suitable needle. This is a very important instrument. The one that I have found the most convenient is made by drawing the temper of a jeweler's broach, No. 6, and repointing on an emery stone. The patient being placed in strong light, the surgeon fixes the lid in a Desmares or Knapp clamp. The patient holds the handle of a positive electrode in the right hand and places the moist sponge on the palm of the left, after the needle is introduced into the hair gland. The needle may be withdrawn after about ten seconds. The patient should remove the sponge from the left hand simultaneous to the withdrawal of the needle.

The number of cells to be used should be decided by the surgeon's knowledge of the condition of his battery. I used from six to ten cells of zinc carbon battery. When the hairs are very fine and obscure, the use of a *three-inch* lens will be found quite serviceable. After electrolysis, the cilia should be removed with epilation forceps. The only objection to the operation in my experience, is that when there is a large number of cilia to be removed, the pain becomes somewhat tedious, though with the clamp I find the pain is not so great. Only about fifteen per cent. of the cilia return. The irritation following the operation is slight. The lids will be swollen for a day or two. In one case, from which I removed but two or three hairs, the operation was followed by the growth of 15 or 20 minute cilia, which were permanently removed.

I have noticed that chelazion and other cystic tumors of the lids would be very rapidly absorbed, when treated from 15 to 20 seconds with the same needle.

My record shows more than fifty cases, and in all, so far as I know, the results were good. In twenty-two cases which have been under observation for more than six months, since the last operation there has been no return of the lashes removed. We have used this operation at the State Eye and Ear Infirmary, and at the Central Free Dispensary, in simple trichiasis; entropium, previously operated on, and but partially successful, leaving a few misdirected hairs sweeping the cornea; also, in cases of entropium which are unfit for operation. In too dense growth of cilia, that sometimes occur in scrofulous children, and interfering with sight.

All diseases which interfere with the refracting media of the eye are of great moment to patient and physician. Among the most important are those affecting the transparancy of the cornea and the regularity of its surface.

